# Finding Priority Areas in the Evaluation of Strategies for the Prevention of Leishmaniasis in an Endemic Municipality of Brazil

**DOI:** 10.3390/tropicalmed9050115

**Published:** 2024-05-16

**Authors:** Talita Carolina Bragança de Oliveira, Anaiá da Paixão Sevá, João Alfredo Biagi Camargo Neto, Uelio de Lima Lopes, Katia Denise Saraiva Bresciani

**Affiliations:** 1 Animal Health and Production Department, Paulista State University (UNESP), Araçatuba 16018-805, Brazil; joao.alfredo@unesp.br (J.A.B.C.N.); katia.bresciani@unesp.br (K.D.S.B.); 2 Department of Environmental and Agrarian Science, State University of Santa Cruz, Ilhéus 45662-900, Brazil; ullopes@uesc.br

**Keywords:** *Leishmania* spp., dog, serology, spatial analysis

## Abstract

Visceral leishmaniasis is a zoonotic disease that affects humans and dogs. The infection is endemic in the municipality of Araçatuba, São Paulo, Brazil. Given the role of dogs in the epidemiology of leishmaniasis, strategies to enhance surveillance and reduce transmission are focused on dogs. In this study, we retrospectively analyzed records of canine visceral leishmaniasis from 2013 to 2022. According to this database, the prevalence of dogs testing positive for leishmaniasis fluctuated, with an average of 65.04% (6590/10,133). Cases were clustered in 10 statistically significant areas. Environmental analyses identified a significant geographical association between animals testing positive and higher vegetation density rates compared with animals testing negative. The period from sample collection to diagnosis and euthanasia, as recommended by the Brazilian Ministry, correlated with disease prevalence and decreased over time. These findings serve to implement different action plans against leishmaniasis for each geographic region and to understand the impact and efforts of strategies in an endemic area.

## 1. Introduction

Visceral leishmaniasis (VL) is of considerable One Health importance due to the fact that it impacts both animals and humans and due to its potentially severe clinical outcomes [1,2]. The high number of cases in certain endemic areas underscores the need for improved diagnostic procedures and control strategies.

VL is endemic in 60 countries [3]. In Latin America, 90% of cases occur in Brazil [4]. In the state of São Paulo, the infection is present in 41.3% (268 out of 648) of the municipalities [5]. The disease was first detected in the state in 1999 when the first autochthonous case were reported in the municipality of Araçatuba, where leishmaniasis is now considered endemic [6].

In Brazil, the causative agent of VL is the protozoan *Leishmania infantum* (*syn. chagasi*). The parasite is transmitted to humans, wild animals, and dogs mainly through the blood meal of an infected sandfly (*Lutzomyia longipalpis*). Dogs are considered important reservoirs for the parasite [7], and the association between human and canine cases in terms of time and space has been reported [8,9,10].

The temporal and spatial distribution of vector-borne diseases is strongly influenced by climatic and environmental variables. Changes in these variables due to human activities, along with the lack of public investment and sanitary infrastructure, favor the occurrence of these diseases [11]. The immature forms of sandflies develop in shaded, moist micro-habitats rich in organic nutrients [12]. Typical breeding sites include garbage dumps, orchards, and chicken coops. All stages of the vector are sensitive to climatic factors, such as temperature and humidity [13]. Given this, the combined study of spatial factors and socio-economic indicators enables the identification of spatial characteristics that favor disease transmission and, hence, risk areas. This helps public health authorities to make better-informed decisions on control and prevention measures [14].

The “Plan of Action to Strengthen the Surveillance and Control of Leishmaniasis in the Americas” (PCPLA) was developed in 2018 by the Pan American Health Organization (PAHO) in collaboration with the Brazilian Ministry of Health (BMH). This plan aims to reduce the incidence and mortality of the infection by 50% by 2022 [15,16]. The progress achieved by this plan is difficult to assess because updated results are not yet available. Despite this Plan of Action, the Brazilian Ministry of Health developed the Visceral Leishmaniasis Surveillance and Control Program (VLSCP) before the year 2000. This program includes the euthanasia of seropositive dogs, among other measures focused on vector control and human treatment. This first measure aims to reduce the number of infected dogs in the environment, thereby also reducing human cases [17]. The number of human cases has been decreasing in Brazil in recent years; however, prevalence of the disease in both humans and dogs remains high in some endemic regions of the country [18,19,20,21,22,23,24,25]. The strategy focused on dogs is being questioned to the extent that new strategies are being reconsidered, implemented, and studied [26,27,28,29].

In this study, we investigated the occurrence of *Leishmania* spp. infections in dogs in Araçatuba, São Paulo, Brazil, and their association with environmental factors and epidemiological characteristics. Thus, we focused on two aspects in this study. In the first one, we conducted a retrospective analysis of the protocol used by the Municipality for controlling leishmaniasis in dogs to prevent the disease in humans, as proposed by the VLSCP. In the second one, we conducted a spatial analysis, along with an ecological analysis of environmental factors influencing the seropositivity rate in dogs. Thus, priority areas for intervention were identified.

## 2. Materials and Methods

### 2.1. Study Area

**Figure 1:**
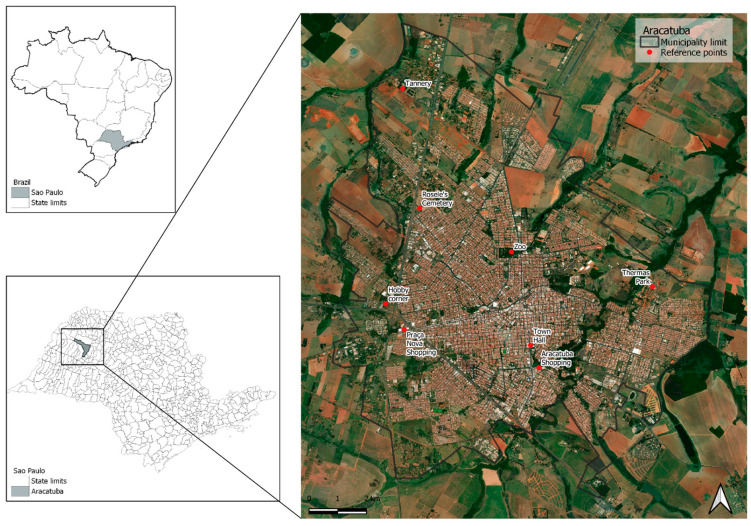
Location of the municipality of Araçatuba in the state of São Paulo, Brazil. The left panels show the location of the state of São Paulo in Brazil and the municipality within the state.

The study area comprised the urban center of the municipality of Araçatuba (Figure 1), located in the State of São Paulo, Brazil, with an estimated population of 194,873 inhabitants [30].

### 2.2. Data of Tested Dogs 

All the data used in the study were obtained from forms that reported the results of field collections conducted between 2013 and 2022 by the Municipal Zoonosis Control Center, pertaining to the investigation of dogs infected with *Leishmania* spp. Canine leishmaniasis was diagnosed during an annual canine seroepidemiological survey in the municipality, which included a range of actions from the Manual for Surveillance and Control of Visceral Leishmaniasis [17] from BMH.

Blood was collected from dogs and centrifuged to obtain serum for the serologic diagnosis of infection by *L. infantum* (*syn. chagasi*). This was conducted in compliance with the latest VLSCP convention. This diagnostic protocol includes a rapid immunochromatographic test (dual-path platform, TR-DPP^®^-LVC) for trial, followed by an enzyme-linked immunosorbent assay (ELISA, Canine Leishmaniasis EIE kit) to confirm positive results from the rapid test. This approach aims to enhance the specificity of both tests. The DPP is a subjective assessment method for testing the anti-*Leishmania* antibody in the *L. donovani* complex using the antigen-like recombinant protein K28 (components K26, K39, and K9) [31]. ELISA uses soluble antigens in the form of promastigotes from parasites such as *Leishmania* major-like (ELISA-L. major like) antigens, offering similar accuracy and reliability in diagnosing *L. infantum* [31]. Both tests were produced by Bio-Manguinhos, Fiocruz, in Rio de Janeiro, Brazil, and used in accordance with the manufacturer’s guidelines.

The diagnostic protocol mentioned above is more specific than sensitive because it is designed for use in euthanizing seropositive animals, with the aim of not leaving any infected hosts (sources of infection) in the environment. Although tests for the molecular identification of parasites in dogs are more accurate, we aimed to identify and prioritize routine practices with practical applicability and understand how they have been conducted. Additionally, this official protocol has generated a large database from the numerous animals sampled in the municipality over an extended period (almost ten years).

The data were entered into an Excel spreadsheet because there was no database containing such information. Additionally, the existing system, FlebWeb, does not provide detailed information. Therefore, the forms were segmented into 3813 municipal blocks, encompassing 107,698 properties, by compiling the results from paper files.

### 2.3. Data Analysis

#### Descriptive Analysis

To conduct the descriptive analyses, we considered the frequency of events along with their 95% confidence intervals and minimum, maximum, median, and mean values, as necessary. We then performed bivariate logistic regression analysis to evaluate the infection in dogs, using it as the dependent variable, with potential associated factors such as age range, sex, and number of animals per residence as independent variables. For classifying the dogs’ ages, the following groups were used: Puppy (<1 year); Young (≥1 and <3 years); Adult (≥3 and <8 years); Senior (≥8 and <12 years); and Geriatric animals (≥12 years). All analyses were conducted using the R program (version 3.6.1), with significance considered at *p* < 0.05.

### 2.4. Analyses of Comparisons

We compared the differences between periods (in days) using two frameworks: (1) from the collection of the blood sample to (a) euthanasia over the years and between years; (b) the performance of the DDP^®^ screening test; and (c) the performance of the confirmatory ELISA and (2) from the performance of the DPP^®^ to the performance of the ELISA. Determining these periods is part of the strategy described in the Manual of VLSCP by the BMH [17]. For these analyses, the Aberdeen–Darling normality test was performed, and a non-normal data distribution was observed. Therefore, the Kruskal–Wallis test was conducted, followed by the Mann–Whitney post hoc test, considering results significant at *p* < 0.05, with Bonferroni correction applied to the *p*-value. These analyses were conducted using the R program (version 3.5.1), specifically with the *nortest*, *rstatix*, and *ggplot2* packages, and were illustrated with boxplots.

Areas with a high prevalence of *Leishmania*-infected dogs (clusters) were identified using scan analysis to pinpoint potential hotspots of frequent *Leishmania* spp. transmission. The residence of each animal was georeferenced using the GeoCode application in Google Sheets online. The statistical method known as spatial scanning was applied using the SatScanTM 9.5 software [32]. The Bernoulli method was used to calculate the prevalence rates among seropositive (cases) and seronegative dogs (controls) within and outside circles of varying sizes. For each potential cluster surrounding each observed animal within various radii, we tested the hypothesis that the relative risk of an animal being positive was higher inside the circle compared to outside, with a significance level of *p* < 0.05. The cluster considered most likely was the one with the highest likelihood ratio value [33,34]. The clusters that were found were represented on the map using QGIS software version 3.4.

The vegetation cover and moisture content in the areas surrounding both infected and uninfected dogs were quantified and compared. A buffer radius of 150 m (representing the average flight range of the fly [35]) was established around both positive and negative animals. In each buffer, we calculated the mean values of the Normalized Difference Vegetation Index (NDVI) and the Normalized Difference Water Index (NDWI). Both variables were calculated from raster images with a resolution of 10 m, obtained from the Imaging Division (DGI) of the Cbers 4 satellite of the Brazilian National Institute for Space Research [36], using the Panchromatic and Multispectral Camera (PAN) 10, covering the period from 05 January 2015 to 10 January 2017. This period was chosen because it represents an average value within the study timeframe. To calculate NDVI and NDWI, the following equations were used:

$$NDVI=\left(V-IV\right)/V+IV$$

$$NDWI=\left(Ve-IV\right)/Ve+IV$$

where (Ve), green band; (V), red band; (IV), infrared band.

The results of these equations range from −1 to 1. The closer the value is to 1, the greater the chlorophyll content or the denser the vegetation is for NDVI, and the higher the moisture level is for NDWI. To calculate the NDVI and NDWI, we used the Raster Calculator, and for determining the mean value within buffers, we used the Zonal Statistics functions of the QGIS software. The mean NDVI and NDWI values of the buffers around animals that tested positive were then compared with those of the buffers around the negative dogs. To this end, the Aberdeen–Darling normality test was applied to the NDVI and NDWI values. Given that the data distribution was non-parametric, the Mann–Whitney test was applied, and a *p*-value < 0.05 was considered significant. As mentioned previously, these analyses were conducted using the R program (version 3.5.1), specifically with the *nortest*, *rstatix*, and *ggplot2* packages, and were illustrated with descriptive boxplots.

## 3. Results

**Figure 2:**
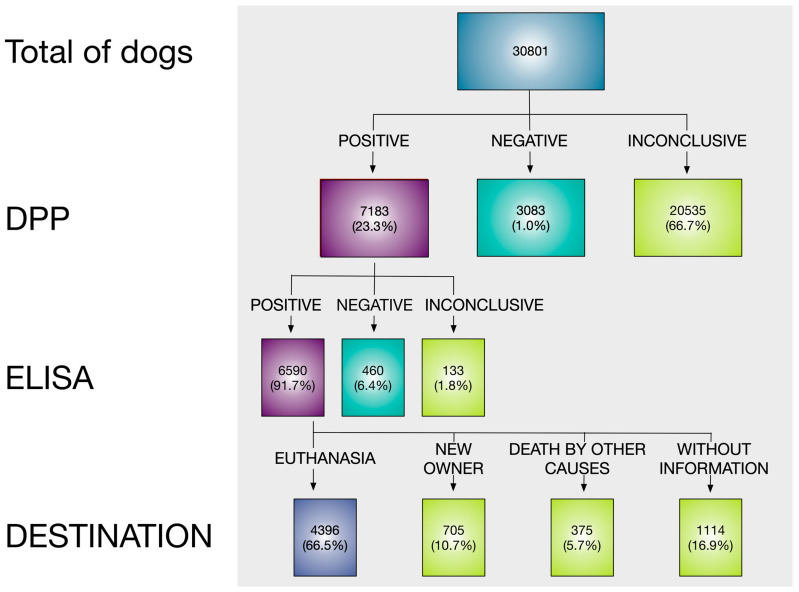
Flowchart of the leishmaniasis survey conducted on 30,801 dogs in the municipality of Araçatuba.

A total of 30,801 animals were evaluated, of which 66.7% (20,535) either did not have their results recorded, were denied participation by the owner, or received an inconclusive *Leishmania* diagnosis. For the remaining 10,266 dogs, a conclusive PPD test result was obtained, revealing that 69.96% (7183/10,266) of them were positive for *Leishmania* (Figure 2). In the confirmatory test (ELISA), 6.52% (133) of the results were declared inconclusive because they were indeterminate or not reported. Among the 7050 results, 93.48% (6590) were positive. Thus, considering all conclusive tests, the prevalence of positive dogs was 65.04% (6590/10,133; 95% CI: 64.11–65.96%), and when considering all sampled animals, 21.4% (6590/30,801) were positive according to both tests.

Most of the owners of *Leishmania* seropositive dogs (66.53%; 4396/6590) agreed with euthanasia, which is an alternative to the BMH protocol. For the remaining seropositive dogs, 14.49% (955) of the owners refused euthanasia, and 10.70% (705) reported that the dogs had fled, were not found, had been given to other people without a possibility of tracking, or the owners had moved. A small number of dogs, 375 (5.69%), had died from other causes.

**Figure 3:**
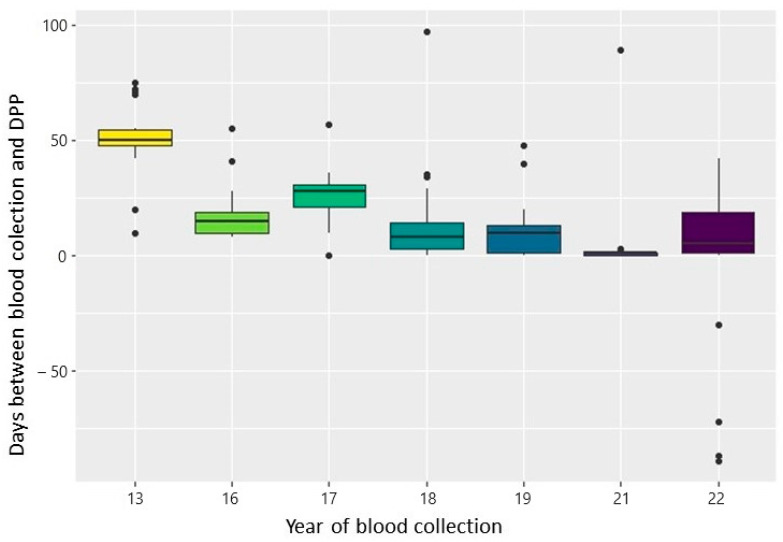
Time interval (in days) between blood collection and the DPP screening test from 2013 to 2022 in the municipality of Araçatuba, São Paulo.

The interval between blood collection and DPP screening decreased significantly from 2013 to 2022, excluding 2014 and 2015 when no data were provided (*p* < 0.001) for each year compared to the previous year, and across all years; Table 1). In 2013, the mean and median time intervals were 53 and 50 days, respectively, while in 2022, the mean and median were 17.8 and 20 days, respectively. There were exceptions in the comparison between 2018 and 2019 (*p* = 0.164, row 16 in Table 1), which showed medians of 8 and 10 days, respectively, and between 2019 and 2022 (*p* = 0.393, row 20 in Table 1), with medians of 10 and 5 days, respectively, as illustrated in Figure 3. The reduction in the time interval in 2021 corresponds to the COVID-19 pandemic and the restricted access of health agents to households [37]. Fewer samples were collected then, allowing for more time to apply the screening test more quickly.

**Figure 4:**
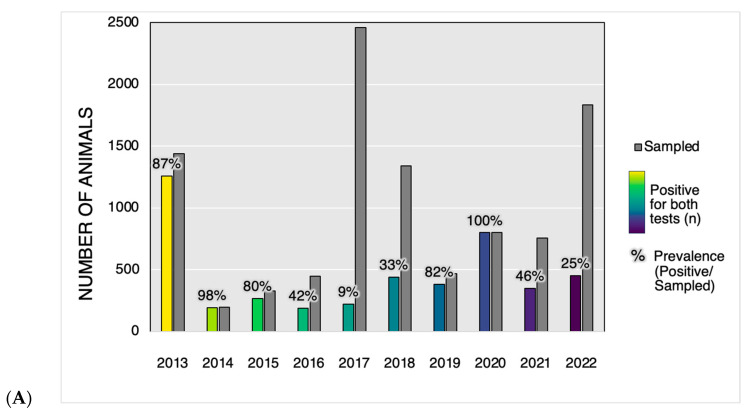

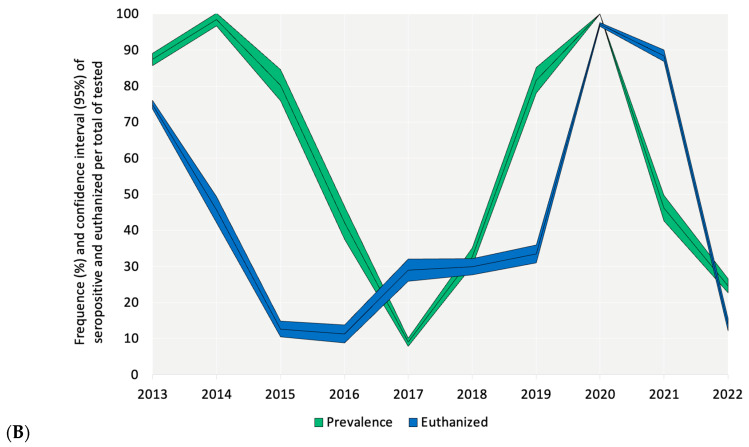
(**A**): Number of samples tested positive for visceral leishmaniasis in both tests and their prevalence (%). (**B**): Comparison between the prevalence (positive for both tests/sampled with conclusive results) and the frequency of euthanasia (euthanized/positive) throughout the year in Araçatuba, São Paulo.

A total of 66.5% (4396) of the seropositive animals (according to both tests) were euthanized, while 171 had no recorded subsequent history. The highest numbers of positive dogs were observed in 2013 (1259) and 2020 (799), in years with the highest prevalence (87% and 100%, respectively) (Figure 4A). The highest percentages of dogs euthanized that tested positive for both tests occurred in the years 2013 and 2020, at 76.0% and 97.6%, respectively (Figure 4B). In the subsequent years (from 2014 to 2017 and from 2021 to 2022), there was a progressive reduction in the number of positive animals, as well as a decrease in the frequency of euthanasia. From 2018 to 2020, the prevalence of positive animals increased progressively compared to 2017, along with the frequency of euthanasia, which reached its maximum in 2020 (100%). After reaching the highest frequency of euthanasia, the prevalence decreased in the following year (2021). Thus, considering a one-year delay between euthanasia and the prevalence of seropositive dogs, the positive correlation was moderate but not significant (r = 0.62; *p* = 0.052). However, when considering euthanasia and prevalence within the same year, the correlation remained positive but weak (r = 0.28; *p* = 0.458). Even with these observations of temporal associations of facts, we must point out that in 2020, due to the COVID-19 pandemic, there was no active search for dogs for VL diagnosis. The only diagnosis at the time came when the owners, noticing their animals were already sick, sought help from the Municipality’s Service Center, where the animals then tested positive for the disease. In the same Center, the animals were euthanized with the owner’s consent, achieving 100% euthanasia coverage.

**Figure 5:**
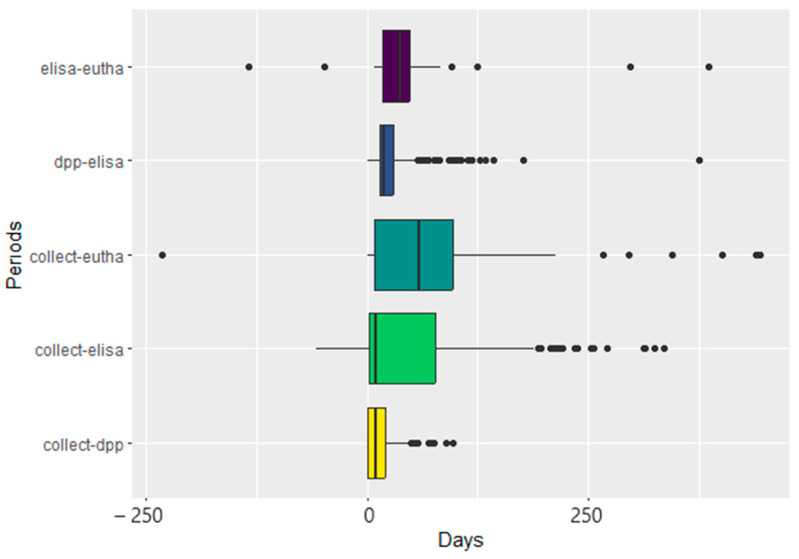
Comparison of the dates for blood collection, screening and confirmatory tests, and euthanasia of dogs in the municipality of Aracatuba, São Paulo, from 2013 to 2022. Periods between blood collection and DPP test (collect–dpp); blood collection and confirmatory test results (ELISA) (collect–elisa); blood collection and euthanasia (collect–eutha); the screening test (DPP) and the confirmatory test (ELISA) (dpp–elisa); and confirmatory test results (ELISA) and euthanasia (elisa–eutha).

The time from blood collection for examination to euthanasia (collect–eutha) was recorded for 1.8% (79/4396) of the euthanized animals, with a maximum duration of 401 days, a median of 49 days, and an average of 68.1 days (Figure 5).

The information on the interval in days from the performance of the ELISA to the date of euthanasia (elisa–eutha) was obtained for 2.30% of the animals (101/4396). However, in 1.98% (2) of the cases, this period was less than zero, indicating that they died before the results of the confirmatory test were disclosed, possibly due to VL or other diseases with a poor prognosis. Excluding these two animals, the interval ranged from 9 to 386 days, with a median of 37 days and a mean of 40.7 days (Figure 5).

The interval between the DPP and ELISA tests was zero days in 8.8% (311) of the samples, meaning the tests were performed on the same day. This rate reflects the optimization of diagnosis. This period had an average duration of 14 days, with a maximum of 91 days (Figure 5).

The prevalence of seropositivity was significantly higher (*p* < 0.001) in adult (8.7%), senior (10.0%), and geriatric dogs (10.5%) than in puppies (4.7%) (Table 2). Therefore, the older the age group, the higher the likelihood of infection, with odds ratios (ORs) of 1.92, 2.28, and 2.39, respectively. Although the prevalence of infection was lower in females than in males (7.6% vs. 9.3%), the association was not statistically significant (*p* = 0.064).

The number of dogs per household was categorized into ranges as shown in Table 2. Households with up to two dogs had a higher prevalence of seropositive dogs, 51.3% (2598), compared to households with more dogs. The prevalence was 76.5% in households with three to four dogs and exceeded 80% in households with more than five dogs, with significant differences (*p* < 0.05) observed between the categories.

**Figure 6:**
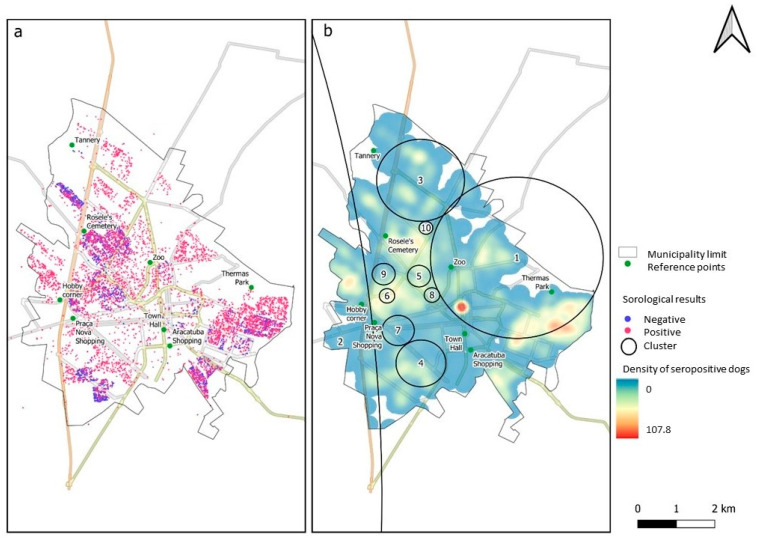
Spatial distribution of dogs sampled and their leishmaniasis serology status from 2013 to 2022 in the urban area of Araçatuba. (**a**) Spatial distribution of households with dogs testing seropositive and seronegative according to the DPP and ELISA tests. (**b**) Clusters of significant prevalence and intensity of seropositive dogs. Legend. Data of cluster are in Table 3.

Seropositive and seronegative animals were observed in all surveyed regions. The distribution of the dogs’ residences, as well as their serological statuses, from 2013 to 2022, is shown in Figure 6.

There were parts of the study area where the spatial prevalence of seropositive dogs was higher than in others, with ten significant clusters of high prevalence (above 80%). Figure 6b shows that in the central region, there are areas with high prevalence, including clusters #7 and #8, where the prevalence reached 100% (with populations of 50 and 37 animals, respectively). Additionally, the risk of infection within these clusters was 1.56 times higher than in areas outside the clusters (Table 3). It should be noted that in the northern part of the region, there was a greater dispersion of sampled animals, and they were found in lower densities. However, a cluster (#3) was observed with a radius of 1.63 km where the risk of infection was 1.63 times higher than the risk outside this area, with a prevalence of 99.3% (444/447). Also in the northern region, cluster #10 had a prevalence of 100% (28) and had the smallest radius among the clusters at 0.26 km. Additionally, it had a high risk of infection, being 1.56 times higher inside than outside. Cluster #2, located in the western region of the municipality, had the largest radius (66.92 km) and a high relative risk of 1.57, along with a prevalence of 97.6% (611/626). Therefore, its risk was associated with the urban area interspersed within the cluster. Cluster #1, located further east, had a radius of 3.20 km. Although it had the lowest prevalence, at 84.2% (1936 out of 2300), it was the largest among the clusters. It is important to note that the Kernel map (heat map) in Figure 6b represents the density of seropositive animals. However, this does not imply that areas appearing redder (hotter) necessarily have a higher prevalence, because areas with a higher number of positive dogs can also have a larger number of negatives. On the other hand, the cluster identified areas of high prevalence, even though within the cluster there might be a low number of dogs, but with a high prevalence of positives. For instance, consider cluster 7, which has a small number of dogs (50), yet a 100% prevalence rate. In this area, the kernel map appears more bluish compared to other areas, similar to cluster 6, which has a smaller radius.

In summary, in the central part of the urban region, clusters with high VL prevalence were identified, but these areas did not necessarily overlap with areas of high dog density. This was also observed in the eastern region of the municipality, where a high density of positive animals was observed, but not high prevalence, because there were also many negative dogs. The cluster located in the western region (2), despite mainly consisting of points with a low density of seropositive animals, had some points with higher density.

**Figure 7:**
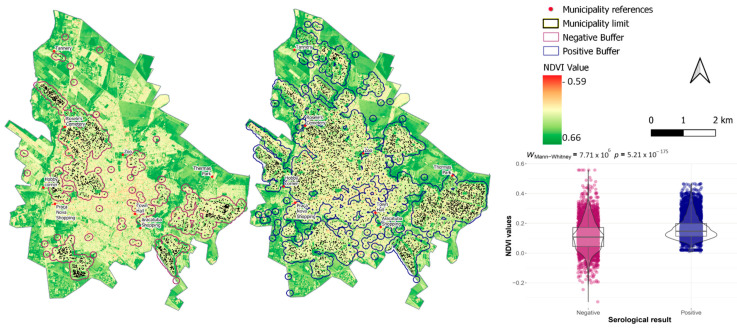
NDVI with locations of seronegative and seropositive dogs, with a buffer of 150 m, for the years 2013 to 2022, in the urban area of Araçatuba. Boxplots are with statistical results of Mann–Whitney analysis.

Regarding NDVI, values closer to 1 indicate denser vegetation. Specifically, values ranging from 0.2 to 0.4 correspond to grass and sparse vegetation, while values from 0.1 to 0.2 primarily indicate exposed soil [38]. In this study, there was a significant difference in NDVI between areas with seronegative and seropositive dogs (U = 7.71 × 10^6^; *p* < 0.001), with mean values of 0.11 (standard deviation = 0.098) and 0.16 (SD = 0.072), and medians of 0.16 (interquartile range—IQR = 0.045–0.172) and 0.15 (IQR = 0.113–0.198), respectively. Thus, although reactive dogs were found throughout the study area, they were more prevalent in places with discreetly higher levels of chlorophyll, while non-reactive animals were more common in areas with greater afforestation or more paved surfaces (Figure 7).

**Figure 8:**
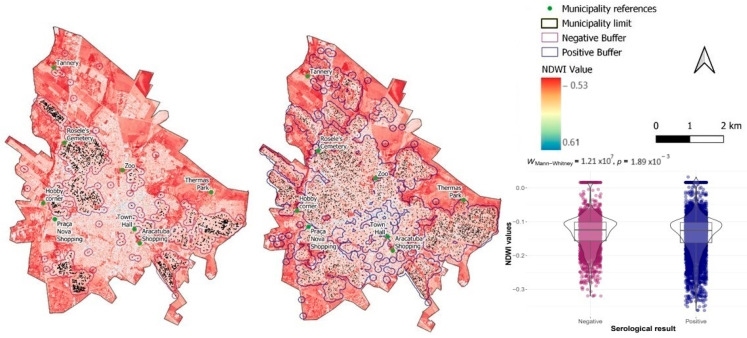
NDWI with locations of seronegative and seropositive dogs, with a 150 m buffer, from 2013 to 2022, in the urban areas of the municipality of Araçatuba, São Paulo. Boxplots are with statistical results of Mann–Whitney analysis.

Regarding NDWI, the mean values for seropositive animals were discreetly lower than for seronegative animals, equivalent to −0.14 (SD = 0.162) and −0.13 (SD = 0.157), respectively. Meanwhile, the medians were −0.125 (IQR = −0.162 to −0.103) and −0.124 (IQR = −0.157 to −0.101), respectively. Although these differences are statistically significant (U = 1.12 × 10^7^; *p* < 0.001), the variations in water at the soil surface are not significantly different (Figure 8). The range between −1 and −0.3 indicates drought or non-aqueous surfaces [38].

## 4. Discussion

A seroprevalence of 21.4% among all animals tested, and 65.4% when only considering conclusive tests, indicates a high prevalence of canine leishmaniasis. During the same period (from 2013 to 2022), the average number of human cases was 6.1 per year, corresponding to an incidence of 3.13 cases per 10,000 inhabitants per year [30].

A few negative ELISA results were expected because the screening test (DPP) has high sensitivity, while the confirmatory test (ELISA) is more specific [39,40]. Although the combination of DDP and ELISA has been questioned [41], their joint use can increase the specificity of the diagnosis and decrease the number of false positives [42], thereby reducing the prevalence of canine reservoirs through euthanasia once the animal considered a reservoir is removed. Regardless of its accuracy, this strategy involving euthanizing positive animals based on the outcome of serological tests to prevent the transmission of leishmaniasis to humans can be questioned on the basis of the high number of inconclusive results as in other studies [43,44] or absence of samples due to the owner’s refusal (67.1%; ((20,535 + 133)/30,801)), in addition to other reasons. Unfortunately, this diagnostic scheme still allows animals that test positive to remain as a source of infection.

Inconclusive results may result from the improper storage of rapid test reagents, the method of conducting the tests, logistical and operational challenges, and the absence of trained personnel to administer the tests and interpret the results. Therefore, it is necessary to improve the accuracy and precision of diagnostic tests.

The results of this study indicate a decrease in the interval between blood collection and testing starting in 2018. This year marked the implementation of the Action Plan in the municipality, which allocated resources to analyze VL and enhance program operations. However, we must consider that not all actions were carried out simultaneously. For instance, the frequency of animal sampling and euthanasia of seropositive individuals fluctuated between 2018 and 2021, showing high rates, but these were not as significant when compared to the years 2013, 2017, and 2022. According to preliminary analyses, Brazil has reduced the incidence of the disease by 50%, although the same reduction was not observed in lethality. However, all countries have made progress compared to initial data, highlighting the efforts of health services in surveillance, control, and assistance [45]. Despite the observed reduction in human cases, it is not necessarily a consequence of efforts to control VL in dogs, especially considering the period includes the years of the COVID-19 pandemic, during which human behavior differed from previous years. To date, there is no study explaining the reduction in human cases during the pandemic. However, it can be assumed that people left their homes less frequently and, therefore, were less exposed to the VL vector. Additionally, some COVID-19 deaths may have been concurrent with VL but were only diagnosed as COVID-19 due to the primary focus on controlling this disease.

The large interval between blood collection, subsequent testing, and the euthanasia of infected animals represents a significant risk [46] due to the continued presence of positive dogs in the community. In our study, although the total number of positive animals euthanized was low (2.3%), the delay period reached 386 days, with a median of more than one month. This delay increases the risk of transmission and reduces the effectiveness of control efforts [42,47,48,49,50]. Thus, the availability of rapid tests and the training of laboratory technicians at the Center for Zoonoses Control allowed for a reduction in this interval, representing an advancement in controlling transmission.

As shown in Figure 5, we expected the period between sample collection and euthanasia to be the longest, as it encompasses the initial and final stages of the process, including screening and confirmatory testing. However, the time between the ELISA test and euthanasia was also prolonged, which may have been due to difficulties in contacting the owner before euthanasia. This could include reasons such as a change of address, which was the case for 10.7% (705) of the animals. Often, this was intentional due to the owner’s resistance to having their animal euthanized.

In conclusion, after analyzing the entire Plan of Action over time, including the intervals between each measure (collecting samples, testing animals, and euthanasia), the number of animals sampled and the percentage of euthanasia for seropositive dogs have improved. Additionally, a positive correlation was observed between the frequency of euthanasia and the prevalence of seropositive animals in subsequent years. This correlation was also observed in other practical and theoretical studies [48,51,52,53,54]. However, there are limited data to support this observation, resulting in a non-significant comparison. It is important to consider that euthanasia in a specific year may involve animals identified as positive in previous years, causing an impact on the next year. As demonstrated, the period between sample collection and euthanasia extended up to 401 days. Despite these issues and the Plan of Action recommended by BMH, the prevalence of dogs in the municipality remains high (24.6% in 2022). Several studies support the notion that these measures need to be applied consistently to achieve significant efficacy. Additionally, they highlight the importance of focusing on other factors, such as vector control and educating dog owners on protecting their animals through preventive measures [52,53,55,56]. This approach is common in endemic regions.

The lack of information on VL for dog owners and other citizens, such as the potential severity in humans, the importance of monitoring the infection in dogs, and the availability of rapid diagnosis for dogs, hampers control efforts, a fact that is evident in Araçatuba [57,58]. Therefore, many owners wait for a confirmed diagnosis before adopting preventive measures, such as using collars impregnated with insecticides. Consequently, the lengthy periods between sample collection and diagnosis can significantly impact the effectiveness of treatment options. Maintaining seropositive animals is a persistent problem that affects the implementation of control strategies [59,60]. In this study, this was revealed by the high number of tests that did not lead to a final diagnosis and the long intervals between the different stages of the strategy. These drawbacks can be attributed to various reasons, such as (1) difficulty in making decisions due to insufficient knowledge about the actual epidemiological situation of VL in dogs; (2) the inadequate storage of diagnostic data for epidemiological surveillance purposes; (3) the collection of irrelevant information or absence of necessary data; (4) a shortage of trained personnel for effective monitoring; and (5) a lack of awareness among the public and professionals regarding the importance of this data [61,62,63,64].

Although a positive correlation was found between the reduction in positive animals and euthanasia coverage, as in other studies, its application is still questionable. The practice of euthanizing asymptomatic infected dogs can have psychological impacts on both dog owners and veterinary professionals [65]. In Brazil, owners typically consider pet dogs to be like children, family members, friends, or companions. Working animals are rare. During the pandemic, more dogs were acquired, mainly by households without children or by people living alone [66]. This shift in pet ownership calls for alternatives to euthanasia. Several studies, both theoretical and practical, have assessed the effectiveness of measures aimed at preventing contact between vectors and dogs or at reducing the population of sandflies [27,28,55,67,68,69,70,71,72,73,74,75]. Additionally, for this reason, the BMH is implementing the use of insecticide-impregnated collars on dogs in endemic municipalities as part of exploring new and more practical strategies to control the disease [29].

A fact associated with resistance to acceptance the euthanasia by dog owners can be greater adherence to treatment. In this regard, the introduction in 2018 of a more specific drug for VL in dogs in Brazil, miltefosine [76], may encourage owners to treat their pets. Although the treatment is expensive, the release of this drug has given the owners more flexibility in their decision making. However, the refusal to euthanize seropositive animals, or even to provide proper treatment, can be considered a risk factor in endemic areas, such as Araçatuba. Although treatment can reduce the parasite load, it is not able to eliminate the parasite from the host completely, instead only promoting clinical improvements. Therefore, other forms of prevention are needed [77]. In this context, treating dogs does not constitute a public health measure for controlling VL but rather a decision made by individual owners [78,79].

Owners often opt for euthanasia when their animals are severely ill and have severe symptoms. Studies have demonstrated that symptomatic patients carry a higher parasite load and have a greater ability to infect vectors compared to potential hosts, among others [80,81,82]. However, studies have shown that the infectivity of asymptomatic and symptomatic animals is similar [83]. It has been reported that approximately 60% of infected animals are asymptomatic [84,85]. The presence of these animals, whether treated or not, serves as a source of infection.

Regarding VL infection and the biological characteristics of the evaluated dogs, we observed that seroprevalence was higher in adult, senior, and geriatric animals than in puppies. Although some studies have demonstrated that puppies are more susceptible, due to their lower immunity [86,87], other studies have found that older dogs, having had more time of exposure to the vector in an endemic area, consequently have a higher risk of contracting *Leishmania* spp. infection [88]. Additionally, it should be considered that the diagnostic tests used identify antibodies, which can persist for long intervals in the body, indicating that the animal had some contact with the parasite [84,89,90]. Thus, the presence of antibodies in older animals may indicate either recent or past infections, while in younger animals, it is more likely to suggest a recent infection.

The presence of more than two dogs in a residence was significantly associated with a higher number of dogs per household. Therefore, we believe that the higher the prevalence of canine cases as source of infection, the higher the abundance of flies, and consequently, the greater the risk of infection [91].

Although the percentage of samples that were either not diagnosed or inconclusive was high (66.67%), the number of samples with conclusive results was also significant (6590). Since the undiagnosed samples appeared to be random, it is very likely that the spatial distribution we observed accurately represents the population. Significant clusters of high prevalence were identified in the northern, central, northeastern, and eastern regions of the city. This observation is not consistent with the decisions usually made by public health authorities, who primarily concentrate their control efforts on the eastern region (personal communication). Therefore, our findings can encourage a shift in the perspective of municipal authorities, leading to a prioritization of neglected areas.

The discovery of clusters with a high prevalence in the study area can aid in the planning of surveillance and control strategies, as implemented in various countries [92,93]. In this study, the clusters were identified based on the presence of VL in dogs. However, we suggest that they represent areas of elevated risk of human infection, since the spread of the disease is related to both space and time between dogs and humans [8,9,10].

We observed that both negative and positive animals were distributed throughout the urban area of the municipality. However, this zoonosis was highly prevalent in certain clusters, with some having a high prevalence of positive animals (>80%). The 10 clusters revealed that Araçatuba has many areas requiring attention, possibly due to socio-economic and environmental factors that contribute to the abundance of vectors and the potential presence of hosts throughout the area. For example, cluster #1 included the municipal emergency room, where the relative risk of contracting the disease was 1.44 times higher than in surrounding areas. This cluster also includes the municipal zoo, which is a large area with several fruit trees and organic material. It is important to mention that the vector of VL (*Lu. longipalpis*) has adapted well to urban environments [94,95].

As reported in other studies [96,97], the NDVI analysis revealed significantly higher values in areas with positive results. This result suggests that the disease was more prevalent in areas with denser vegetation. Negative results were associated with areas with less vegetation. In the NDWI findings, higher values of this index are associated with a greater presence of biomass and photosynthetically active elements [87]. The number of cases is influenced by environmental and climate factors, as the vectors are sensitive to these factors [98,99]. In the area, there are rivers and streams, and despite the significant difference indicating a higher prevalence of positive animals in drier areas, these values represent almost the same level of humidity, with medians equivalent to −0.125 and −0.124 for seronegative and seropositive animals, respectively.

Based on observations of canine cases, there is a need to develop new strategies and prioritize surveillance and control programs to reduce the incidence of VL in humans. In the city of Araçatuba, the presence of chlorophyll was associated with positive cases, and several clusters were identified. However, in other municipalities, the distribution of cases may vary due to environmental and socio-economic factors, or characteristics of the canine population. It is also necessary to enhance the use of spatial tools and incorporate environmental variables to identify clusters at a high risk of transmission and potential associated factors. This type of analysis can be applied to other regions and adapted for different time periods to help health authorities target strategies towards specific areas within endemic municipalities.

## 5. Conclusions

We observed that the seroprevalence of VL in dogs in the municipality of Aracatuba was high, fluctuated throughout the year, and had an average exceeding 50% when considering conclusive tests over the entire study period. It remained high in the last year (2020). The VL infection rates increased with the age of the animals and the number of dogs present in the households. The extent of canine diagnostic sampling, euthanasia rates, and the intervals between them varied significantly over the years, as officially recommended control strategies were implemented, demonstrating a lack of consistent action within the municipality. Areas with a high prevalence of seropositive dogs, compared to those with fewer, were identified, highlighting the risk of transmission and warranting greater attention from health authorities. The analysis of the biological characteristics of infected animals, strategies for control over time, and the geographic distribution of VL in animals, and in humans, if possible, can help in understanding the behavior of cases and in identifying the efficacy of applied measures, thereby guiding future planning.

## Figures and Tables

**Table 1:** Comparison of the time interval (in days) between blood collection and the screening test (DPP) across all yearly pairings from “A” to “B” (2013 to 2022) in the municipality of Araçatuba, São Paulo, including frequencies and statistical results.

Comparison Number	Year (A)	n (A)	Year (B)	n (B)	Median	Statistical Value (T_s_)	Adjusted *p* Value
1	2013	218	2016	230	50/20	48,672	**<0.001**
2	2013	218	2017	312	50/28	65,174.5	**<0.001**
3	2013	218	2018	872	50/10	185,140.5	**<0.001**
4	2013	218	2019	99	50/15	21,464	**<0.001**
5	2013	218	2021	436	50/3	94,830	**<0.001**
6	2013	218	2022	1372	50/8	295,944.5	**<0.001**
7	2016	230	2017	312	20/28	13,455	**<0.001**
8	2016	230	2018	872	20/10	146,934	**<0.001**
9	2016	230	2019	99	20/15	17,011	**<0.001**
10	2016	230	2021	436	20/3	100,050	**<0.001**
11	2016	230	2022	1372	20/8	209,675	**<0.001**
12	2017	312	2018	872	28/10	246,174	**<0.001**
13	2017	312	2019	99	28/15	29,122	**<0.001**
14	2017	312	2021	436	28/3	135,056	**<0.001**
15	2017	312	2022	1372	28/8	364,856.5	**<0.001**
16	2018	872	2019	99	10/15	50,176	0.164
17	2018	872	2021	436	10/3	369,540.5	**<0.001**
18	2018	872	2022	1372	10/8	658,831	**0.001**
19	2019	99	2021	436	15/3	35,423	**<0.001**
20	2019	99	2022	1372	15/8	58,417	0.393
21	2021	436	2022	1372	3/8	87,374	**<0.001**

Legend. n: number of DPP tests. WC: Value of the Wilcoxon statistic. *p*-values in bold: significant (<0.05), the *p*-values are adjusted with the Bonferroni correction.

**Table 2:** Logistic regression analysis of *Leishmania* seropositive and negative animals.

		**Negative**		**Positive**			**Odds Ratio**	***p* Value**
		**n**	**(%)**	**n**	**(%)**	**Total**	**(CI 95%)**	
Age rate	Puppy	224	95.3	11	4.7	235	reference	
	Young	1066	92.1	91	7.9	1157	1.7 (0.89, 3.23)	0.107
	Adult	1326	91.3	126	8.7	1452	1.92 (1.02, 3.62)	0.043
	Senior	368	90.0	41	10.0	409	2.28 (1.15, 4.53)	0.018
	Geriatric	196	89.5	23	10.5	219	2.39 (1.14, 5.03)	0.022
Sex	Female	2078	92.4	172	7.6	2250	reference	
	Male	1511	90.7	155	9.3	1666	1.24 (0.99, 1.56)	0.064
Number of dogs per residence	Up to 2	2598	51.3	2470	48.7	5068	reference	
	From 3 to 4	819	76.5	251	23.5	1070	0.32 (0.28, 0.37)	<0.001
	From 5 to 7	209	87.1	31	12.9	240	0.16 (0.11, 0.23)	<0.001
	From 8 to 15	22	84.6	4	15.4	26	0.19 (0.07, 0.56)	0.002

Legend. n: number of animals; CI: confidence interval.

**Table 3:** Features of local cluster analysis of seropositive dog prevalences, using spatial scan statistics, are represented in Figure 6b.

Cluster Number	Prevalence (%)	Population (n)	Positive (n)	Relative Risk	*p*-Value	Radius (Km)
1	84.2	2300	1936	1.44	<0.001	3.20
2	97.6	626	611	1.57	<0.001	66.92
3	99.3	447	444	1.63	<0.001	1.63
4	98.9	272	269	1.56	<0.001	0.93
5	97.6	125	122	1.53	<0.001	0.43
6	97.6	84	82	1.53	<0.001	0.29
7	100	50	50	1.56	<0.001	0.60
8	100	37	37	1.56	<0.001	0.29
9	96.1	51	49	1.50	0.0021	0.43
10	100	28	28	1.56	0.032	0.26

## Data Availability

Data are contained within the article.
